# Nanostructured Lipid Carriers to Mediate Brain Delivery of Temazepam: Design and In Vivo Study

**DOI:** 10.3390/pharmaceutics12050451

**Published:** 2020-05-14

**Authors:** Nermin E. Eleraky, Mahmoud M. Omar, Hemat A. Mahmoud, Heba A. Abou-Taleb

**Affiliations:** 1 Department of Pharmaceutics, Faculty of Pharmacy, Assiut University, Assiut 71526, Egypt; 2 Department of Pharmaceutics and Industrial Pharmacy, Deraya University, Minia 61768, Egypt; mahmoudmomar@hotmail.com; 3 Department of Pharmaceutics and Clinical Pharmacy, Faculty of Pharmacy, Sohag University, Sohag 82524, Egypt; 4 Department of Clinical Oncology and Nuclear Medicine, Assiut University, Assiut 71526, Egypt; hemat.ahmad2012@yahoo.com; 5 Department of Pharmaceutics and Industrial Pharmacy, Faculty of Pharmacy, Nahda University (NUB), Beni-Suef 62511, Egypt; habhob61076@yahoo.com

**Keywords:** lipid nanoparticles, brain delivery, nanostructured lipid carriers, factorial design

## Abstract

The opposing effect of the blood–brain barrier against the delivery of most drugs warrants the need for an efficient brain targeted drug delivery system for the successful management of neurological disorders. Temazepam-loaded nanostructured lipid carriers (NLCs) have shown possibilities for enhancing bioavailability and brain targeting affinity after oral administration. This study aimed to investigate these properties for insomnia treatment. Temazepam-NLCs were prepared by the solvent injection method and optimized using a 4^2^ full factorial design. The optimum formulation (NLC-1) consisted of; Compritol^®^ 888 ATO (75 mg), oleic acid (25 mg), and Poloxamer^®^ 407 (0.3 g), with an entrapment efficiency of 75.2 ± 0.1%. The average size, zeta potential, and polydispersity index were determined to be 306.6 ± 49.6 nm, −10.2 ± 0.3 mV, and 0.09 ± 0.10, respectively. Moreover, an in vitro release study showed that the optimized temazepam NLC-1 formulation had a sustained release profile. Scintigraphy images showed evident improvement in brain uptake for the oral ^99m^Tc-temazepam NLC-1 formulation versus the ^99m^Tc-temazepam suspension. Pharmacokinetic data revealed a significant increase in the relative bioavailability of ^99m^Tc-temazepam NLC-1 formulation (292.7%), compared to that of oral ^99m^Tc-temazepam suspension. Besides, the NLC formulation exhibited a distinct targeting affinity to rat brain. In conclusion, our results indicate that the developed temazepam NLC formulation can be considered as a potential nanocarrier for brain-mediated drug delivery in the out-patient management of insomnia.

## 1. Introduction

The blood-brain barrier (BBB) has a preventive effect against the permeation of most therapeutics across the brain, thus limiting the effective management of most neurological diseases [1]. The restrictive nature of BBB is due to its complex structure, which includes several cells (endothelial cells, perivascular mast cells, pericytes, and astroglia). Additionally, the tightness of the BBB inhibits the paracellular transport of most drugs [2,3,4]. The therapeutic targeting of the brain will, therefore, add numerous benefits through a rational approach.

**Figure 1:**
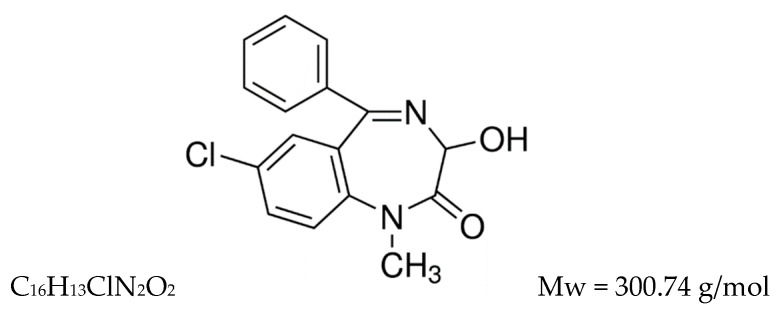
Chemical structure of temazepam.

Temazepam (7-chloro-3-hydroxy-1-methyl-5-phenyl-3*H*-1,4-benzodiazepin-2-one), (Figure 1), is a gamma-aminobutyric acid (GABA) modulator. It is widely used clinically because of its anxiolytic, sedative, and anticonvulsant effects [5,6,7]. Temazepam is classified in the biopharmaceutical classification system as BCS class II, with high permeability and very poor aqueous solubility. The oral bioavailability of temazepam is affected by its poor aqueous solubility and its tendency for inactive hydrolysis in the highly acidic gastric fluid, which precludes its pharmacological effects [8]. Additionally, the lipophilic nature of temazepam limits its ability to partition within the aqueous environment of brain interstitial fluid and thus become entrapped within the capillary bed [9]. Besides, temazepam is highly bound to plasma proteins (96%), limiting its ability to diffuse from the systemic circulation into brain tissue [10]. Consequently, temazepam fails to attain therapeutic concentrations in the brain when administered via the oral route.

To improve the delivery of temazepam, intravenous, rectal, and intranasal administration routes have been investigated. However, due to their limited effectiveness or safety concerns, such approaches have been unsuccessful. For example, intravenous administration requires the use of co-solvents that cause pain during injection and result in a high incidence of venous thrombosis [11]. Rectal and intranasal administrations are also subject to restrictions of low patient compliance, variable bioavailability, and limited adsorption surface, thus lowering the potential to reach the brain with appropriate drug levels [12,13].

To date, very inadequate formulation strategies have tested to improve the dissolution of temazepam and hence provide a more convenient oral drug administration. These include; elixir formulation [14], oral dispersible tablets [15], and temazepam-PEG6000 solid dispersions [16]. Many of these techniques may have improved the solubilization and absorption of the drug. However, they did not show any superior effects in targeting the blood-brain barrier.

Various invasive strategies have been explored to allow therapeutics to overcome BBB. These strategies include; osmotic disruption of the BBB [17], chemical modifications of drugs, “Trojan horse” receptor-mediated transport technique, or the binding of BBB-impermeable drugs to permeable molecules [18]. Alternative routes of administration that cross the brain and escape the BBB (e.g., intranasal) have also been examined [19,20]. Given these premises, it is conceivable to explore a new delivery system that will enable temazepam to overcome BBB through a noninvasive strategy.

Nanoparticles (NPs) technology has recently been introduced to improve drug transit across the BBB [21,22]. Lipid nanocarriers, such as nanostructured lipid carriers (NLCs), are the second class of solid lipid nanoparticles (SLNs). NLCs are made up of solid lipids mixed with liquid oil. This combination results in the formation of a less ordered lipid matrix with improved drug loading efficiency and reduced tendency for drug ejection during storage [23,24]. NLCs have the advantage of enhanced drug transport across BBB, either through passive diffusion or by transcellular permeation across the BBB [25]. Further, lipid nanoformulations can reduce the characteristics of delayed and incomplete dissolution of lipophilic drugs, by forming solubilized phases from which drug absorption can readily occur [26].

Previous studies have shown the enhanced brain delivery of different therapeutics using NLC formulations. For example, improved brain and tumor targeting efficacy was reported for curcumin loaded NLC formulation [27]. Itraconazole loaded NLC formulation also showed a higher concentration of drug in the brain (2-fold) compared to the un-loaded drug [28]. The formulation of baicalein-NLC also resulted in a higher accumulation of baicalein in the cerebral cortex and brain stem compared to the aqueous drug solution [29].

The present research aims to study the effectiveness of using nanostructured lipid carriers as a drug delivery strategy to enhance the bioavailability of temazepam, as well as its brain targeting efficiency following oral administration. Temazepam NLCs were developed using the solvent injection method and statistically optimized using a 4^2^ full factorial design, taking into account process variables such as stabilizer concentration (%*w*/*v*) and liquid lipid type. The prepared formulations were characterized in terms of particle size, polydispersity index (PDI), zeta potential (ZP), and percent entrapment efficiency (%EE). An in vitro release study of the optimized formulation was conducted using a modified dialysis membrane diffusion technique. Additionally, an in vivo biodistribution study was performed on Sprague–Dawley rats to evaluate oral bioavailability and brain affinity of the developed ^99m^Tc-temazepam NLC oral formulation versus ^99m^Tc-temazepam oral suspension. To the best of our knowledge, this is the first attempt to enhance the oral delivery of temazepam through encapsulation into a nanostructured lipid carrier-based delivery system.

## 2. Materials and Methods

### 2.1. Materials

Temazepam was obtained from Sigma-Aldrich (St. Louis, MO, USA). Capryol^®^ 90, Labrasol^®^, and Compritol^®^888 ATO were kind gifts from Gattefossée (Saint-Priest Cedex, France). Miglyol^®^ 840 was obtained from Cremer Oleo GmbH & Co. KG (Hamburg, Germany). Poloxamer^®^ 407 was purchased from BASF (Greenville, OH, USA). Oleic acid was obtained from Alpha Chemicals Co., Cairo, Egypt. Spectra/Por^®^ dialysis membrane, 12,000–14,000 molecular weight cut off, was obtained from Spectrum Laboratories Inc. (Rancho Dominguez, CA, USA). All other chemicals were of analytical grade and used without further purification.

### 2.2. Animals

The animal study was conducted in line with the Animal Ethical Guidelines for Investigations in Laboratory Animals and was approved by the Ethical Review Board of Faculty of Pharmacy, Nahda University, Beni-suef, Egypt (Ref: NUB 010-019, 20 February 2019). Sprague–Dawley rats (male 2–3 months) weighing 150–200 g were obtained from the Central Animal House Facility of Assiut University, Assiut, Egypt. All animals were fed regularly, with access to filtered drinking water. Rats were kept at a room temperature of 25 ± 5 °C.

### 2.3. Fabrication of Temazepam-Loaded Nanostructured Lipid Carriers

Temazepam was loaded within NLCs using the solvent injection method previously described by Chen et al. [30], with the following modifications. Briefly, the lipid phase was prepared by dissolving temazepam (6 µg/mL) and a 100 mg of a Compritol^®^888 ATO (solid lipid) and liquid lipid (oleic acid, Labrasol^®^, Capryol^®^ 90 or Miglyol^®^ 840), (3:1 *w*/*w*) mixture, in 2 mL of acetone/ethanol (3:1 *v*/*v*) at 60 °C. The aqueous phase was composed of (30 mL) distilled water in the presence of Poloxamer^®^ 407 (1, 2, 3, or 5%) as a stabilizer, was heated to the same temperature. The lipid phase was rapidly injected into the aqueous phase. The obtained dispersion was stirred (1000 rpm) for three hours at room temperature, utilizing a magnetic stirrer to enable acetone evaporation. Subsequently, the NLC suspension was sonicated for 20 min. As a control, temazepam-free plain NLCs were fabricated using the same protocol.

### 2.4. Experimental Design

Temazepam loaded NLC formulations were fabricated employing a 4^2^ full factorial design model. The determining factors and levels are described in Table 1. The investigated variables were; stabilizer concentration (%*w*/*v*) (A) and liquid lipid type (B). Each variable was used in four levels, which were specified based on preliminary studies. The measured responses were entrapment efficiency (R_1_), particle size (R_2_), PDI (R_3_), and zeta potential (R_4_).

#### Optimization of Data

Polynomial equations resulted from applying the experimental layout were used to optimize the levels of experimental variables (A and B) to get optimum response values of R_1_, R_2_, R_3,_ and R_4_. An optimized formulation was prepared according to the predicted levels of A and B. The responses were measured and compared with the predicted values.

### 2.5. Characterization of Temazepam-Loaded Nanostructured Lipid Carriers

#### 2.5.1. Particle Size and Zeta Potential

The particle size distribution of the fabricated temazepam-NLCs was determined at 25 °C by dynamic laser light scattering (DLS) technique using the Malvern Zetasizer Nano-ZS (Malvern Instruments, Worcestershire, UK), equipped with a 4 mW helium/neon laser operating at (λ = 633 nm) and a thermoelectric temperature controller. Before each measurement, the NLC suspension was vortexed for at least one minute. Particle size values reported in this study correspond to hydrodynamic diameters. The zeta potential of NLC suspension was measured using electrophoretic mobility data acquired in distilled water [31]. Measurements were performed in triplicate.

#### 2.5.2. Encapsulation Efficiency

The encapsulation efficiency (EE%) of temazepam within NLCs was calculated indirectly by measuring unincorporated temazepam spectrophotometrically using (Shimadzu, model UV-1601 PC, Kyoto, Japan), at λ = 232 nm, after ultrafiltration of temazepam NLCs suspension using (Amicon^®^ Ultra-4 30k Merck Millipore, Fisher Scientific, Pittsburgh, PA, USA) at (4000× *g*) for 10 min.

Calculations were performed according to:

$$\mathrm{EE}\%=\frac{\left[\left(\mathrm{WT}-\mathrm{WF}\right)\right]}{\mathrm{WT}}\times 100\%$$

where WT = weight of total drug added, and WF = weight of free un-entrapped drug quantified in the ultrafiltrate, respectively.

#### 2.5.3. Morphology

Optimized temazepam nanostructured lipid carrier (NLC-1) suspension was imaged using a transmission electron microscope (Hitachi-H7650, Santa Clara, CA, USA). The sample was treated on copper grids pre-wetted with 0.1% (*w*/*v*) BSA. Then, the sample was left to dry for 5 min, followed by washing and staining the adsorbed sample with 2% (*w*/*v*) uranyl acetate. Transmission electron microscopy was conducted at 80 kV accelerating voltage and data acquisition was done using the AMT-700 capture image camera (Advanced Microscopy Techniques, Woburn, MA, USA).

#### 2.5.4. Fourier Transform Infrared (FT-IR) Spectroscopy

The chemical characteristics of temazepam powder, Compritol^®^888 ATO powder, placebo NLC freeze-dried powder, and optimized temazepam nanostructured lipid carrier (NLC-1) freeze-dried powder were evaluated using Digilab spectrum Excalibur FTIR, with an attenuated total reflectance (ATR) Accessory. The dry sample was pressed onto the ATR crystal and the infrared spectrum was recorded from 4000 to 400 cm^−1^.

#### 2.5.5. Differential Scanning Calorimetry (DSC)

The DSC thermograms were obtained using a DSC-50 differential scanning calorimeter (Shimadzu, Seisakusho Ltd., Kyoto, Japan). (1–2 mg) samples of temazepam powder, Compritol^®^888 ATO powder, placebo NLC freeze-dried powder or optimized temazepam nanostructured lipid carrier (NLC-1) freeze-dried powder were placed in aluminum pans and heated at a scanning rate of 10 °C/min from 30 to 250 °C in the presence of nitrogen, at a flow rate of 40 mL/min. By applying DSC-T50 software 1.01 (Shimadzu, Japan), melting point, enthalpy (ΔH, Joule/g), and onset temperature can be calculated.

#### 2.5.6. X-ray Diffraction (XRD)

The crystalline properties of temazepam powder, Compritol^®^888 ATO powder, placebo NLC freeze-dried powder and optimized temazepam nanostructured lipid carrier (NLC-1) freeze-dried powder were assessed using X-ray diffraction by Philips diffractometer (PW-1050, Bragg-Brentano); under Cu K α radiation (35 Kv, 40 mA, slit 1.5418 A°).

#### 2.5.7. In Vitro Release Study

The dialysis membrane diffusion technique [32,33,34] was applied to analyze the in vitro release of optimized temazepam NLC formulation (NLC-1) compared with temazepam suspension. PBS (pH 7.4, 40 mL) was selected as the release vehicle. An accurately weighed amount of freeze-dried temazepam-loaded NLC-1 dispersion in PBS, pH 7.4, equivalent to (141 µg/mL) temazepam was added to a glass cylinder fitted at its lower end with presoaked cellulose membrane (Spectra/Por^®^ dialysis membrane, M.W. cut off 12,000–14,000). The glass cylinder was then immersed in a beaker containing the release medium at 37 ± 0.5 °C and agitated at a constant speed of 100 rpm. Aliquots (3 mL) were withdrawn and substituted with fresh medium. The drug content was determined spectrophotometrically using Shimadzu, model UV-1601 PC (Kyoto, Japan), λ = 232 nm, at predetermined time intervals for 24 h. The release experiment was performed in triplicate. Various mathematical models were applied, in order to determine the kinetics and mechanism of drug release from the developed NLC formulation.

### 2.6. In Vivo Study

#### 2.6.1. Radiolabeling of Temazepam and NLC Formulation with Technitium-99m

The direct labeling technique of temazepam was applied in this study [35,36,37]. Technitium-99m (^99m^Tc) was obtained by elution from molybdenum-99 (Mo-99) to ^99m^Tc generator. Drug solution (3 mg/500 μL temazepam in acetone) was mixed with stannous chloride dihydrate solution (200 μL, 2 mg/mL in ethanol). For the reduction of Tc-99m, the resultant mixture was mixed with 500 μL of free ^99m^Tc eluate (2 mCi) and incubated at 37 ± 0.5 °C for 30 min. NLC formulation was prepared following the same procedure using Tc-99m labeled temazepam. Stable radiolabeled ^99m^Tc-temazepam suspension and ^99m^Tc-temazepam NLC-1 preparation then underwent gamma scintigraphy and biodistribution studies.

#### 2.6.2. Gamma Scintigraphy Imaging

Gamma scintigraphy imaging was performed to provide direct evidence on the exact location of radiolabeled ^99m^Tc-temazepam suspension and optimized ^99m^Tc-temazepam NLC-1 formulation within the body of experimental rats. A total of twelve rats (weighing between 150 and 200 g each) divided into two groups (6 rats per each group) were utilized in this experiment. Then, ^99m^Tc-temazepam suspension or ^99m^Tc-temazepam NLC-1 formulation were orally administered to rats of the respective group through an oro-gastric tube. Using the intra-peritoneal injection of ketamine hydrochloride (0.5 mL, 50 mg/mL), rats were anesthetized and stabilized on the imaging table. At predetermined time intervals (0.5, 1, 2, 4, and 6 h’ post-administration), static images of the whole body were obtained in the posterior projection. Single-photon emission/computed tomography (SPECT/CT) images were acquired to produce three-dimensional images at 0.5 and 2 h for a more precise localization of radioactivity. Static and SPECT/CT images were captured with the help of a SPECT/CT scanner (Symbia T2, Siemens, Munich, Germany) equipped with low energy all-purpose (LEAP) collimator. Planar images were acquired for 10 min. The parameters of SPECT images were (128 × 128 matrix, non-circular orbit, step and shoot, 32 image number).

#### 2.6.3. Biodistribution Study

A biodistribution study was conducted on the same groups of rats (6 rats per each group) after acquiring gamma scintigraphy images. Blood samples were withdrawn from rats after (0.5, 1, 2, 4, and 6 h) through retro-orbital plexus and placed in tubes containing heparin. Plasma was separated from the blood after centrifugation at 3500 rpm for 15 min. Radioactivity was measured using shielded well type gamma scintillation counter (Atomlab^TM^, 500 dose calibrator, wipe test counter).

The in vivo brain distribution study was conducted on 30 rats, each being 150–200 g in weight. The rats were split up into two groups. The first group (3 rats per each time point) received oral ^99m^Tc-temazepam NLC-1 formulation. The radioactive dose each rat administered was (77.5 μCi/10 mL) containing 116.25 µg temazepam (equivalent to 0.775 mg/kg rat dose). Similarly, the second group (3 rats per each time point) received oral ^99m^Tc-temazepam suspension (77.5 μCi/10 mL). The animals were sacrificed at the predetermined time intervals (0.5, 1, 2, 4, or 6 h). The brain was harvested, washed twice with normal saline, and weighed. The radioactivity within brain samples was determined using a shielded well type gamma scintillation counter. Data were reported as a percentage of injected dose per gram (% id/g) according to the following equation;

% id/g=RadioactivitySample wtInjected dose×100

The radiopharmaceutical uptake per gram in the brain/blood was calculated. The pharmacokinetic parameters, including; area under the concentration-time curve (AUC), peak concentration (*C*_max_), time to reach peak concentration (*T*_max_), half-life (*t*_1/2_), mean residence time (MRT) and plasma clearance (CL) were calculated by non-compartmental analysis using PK solver add-in program in Microsoft excel.

To evaluate the brain-targeting efficiency of the developed temazepam NLC formulation, the drug-targeting index (DTI) was calculated according to the following equation;

DTI =(AUC brainAUC plasma) Tem−NLC formulation(AUC brainAUC plasma) Tem−suspension

The relative rates of uptake (Re) and the ratio of peak concentration (Ce), were calculated using the following equations:

Re=(AUC brain) Tem−NLC formulation(AUC brain) Tem−suspensionCe=(Cmaxbrain) Tem−NLC formulation(Cmaxbrain) Tem−suspension

The relative bioavailability of the developed temazepam NLC oral formulation was described based on the following equation;

$$\mathrm{Relative}\;\mathrm{bioavailability}\;\left(\%\right)=\frac{\left(\mathrm{AUC}\;\mathrm{plasma}\right)\;\mathrm{Tem}-\mathrm{NLC}\;\mathrm{formulation}}{\left(\mathrm{AUC}\;\mathrm{plasma}\right)\;\mathrm{Tem}-\mathrm{suspension}}\times 100\%$$

### 2.7. Statistical Analysis

All experiments were performed in at least three independent experiments, and the results are reported as mean ± standard deviation (SD). A statistically significant difference (*p* < 0.05) between treatment groups was assessed utilizing one-way analysis of variance (ANOVA) with Tukey Kramer multiple assessments or two-sided Student’s *t*-test for pairwise comparison (GraphPad Prism 6.0, GraphPad, San Diego, CA, USA).

## 3. Results and Discussion

To improve the oral bioavailability of temazepam and facilitate temazepam brain transport, the main objective of this research was to augment the encapsulation efficiency of this lipophilic benzodiazepine drug within NLCs. It was hypothesized that NLCs containing high temazepam content are transported across the intestinal mucosa, thereby significantly increasing the fraction absorbed (i.e., enhanced oral bioavailability). Simultaneously, the small hydrodynamic diameter and lipophilic nature of NLC formulation enable better contact with BBB and improved brain uptake. In this regard, the experimental design has been widely applied in pharmaceutical development. The models generated for the significant factors are capable of identifying how they influence the critical quality attributes (CQAs) of the formulations, as well as identifying the interactions between the factors that allow the system to be optimized effectively.

### 3.1. Fabrication and Optimization of Temazepam Loaded NLCs

In an attempt to design an efficient NLC delivery system with improved brain targeting capability, the process started with the proper selection of solid lipids, liquid lipids, and stabilizers. Preliminary experiments have been carried out using varied solid lipids (Precirol^®^ ATO 5, Compritol^®^ 888 ATO, and stearic acid), liquid lipids (olive oil, oleic acid, glycerol, Capryol^®^ 90, Labrasol^®^, lecithin, Miglyol^®^ 810, and Miglyol^®^ 840) and surfactants (Poloxamer^®^ 407, Poloxamer^®^ 188, PVA, lecithin, and span 60), in addition to different organic/aqueous phase ratios (1:5 to 1:20 *v*/*v*), data not shown.

It was observed that the concentration of surfactant (A) and the type of liquid lipid (B) exert a noteworthy influence on encapsulation efficiency (EE%), particle size, polydispersity index (PDI), and zeta potential values. Hence, both factors were selected for subsequent studies on the systematic formulation development of NLCs.

Data collected for each response were analyzed utilizing Design-Expert^®^ software (version 12.0.0, Stat-Ease Inc., Minneapolis, MN, USA). The software chooses the model which best fits the data and offers polynomial Equations (1)–(4), in the form of coded factors. These equations are utilized for results extraction after considering the magnitude and sign of the coefficients. A positive sign referred to synergism, whereas a negative sign points out antagonism. Statistical analysis using ANOVA revealed that the sequential model suggested for evaluating the different parameters is cubic. For satisfying the ANOVA assumption, the Box–Cox plot of particle size and PDI recommended inverse square root and square root response transformation, respectively. Residual analysis and ANOVA emphasized the fitting of the underlying models with predicated R-squared values in a good agreement with the adjusted ones. The adequacy/precision ratios for the four dependent variables are higher than 4, denoting the signal’s ability to navigate the space.

(1) $$\mathrm{EE}\;\%=+56.29+18.83A-32.67B+15.14\mathrm{AB}+{1.23A}^{2}+{16.70B}^{2}+{6.44A}^{2}B-{9.76\mathrm{AB}}^{2}+{6.45A}^{3}+{20.31B}^{3}$$

(2) 1Sqrt (P.size)=+0.035−0.013A+0.0068B−0.0026AB+0.0002A2+0.0084B2−0.0055 A2B+0.0048AB2+0.0033A3−0.0111B3

(3) $$\mathrm{Sqrt}\left(\mathrm{PDI}\right)=+0.47-0.25A-0.60B-0.12\mathrm{AB}-{0.054A}^{2}+{0.27B}^{2}+{0.23A}^{2}B+{0.14\mathrm{AB}}^{2}+{0.30A}^{3}+{0.57B}^{3}$$

(4) $$\mathrm{ZP}=-0.65-3.02A+0.60B-0.61\mathrm{AB}-{0.82A}^{2}-{3.59B}^{2}-{2.62A}^{2}B+{1.37\mathrm{AB}}^{2}+{4.87A}^{3}+{3.64B}^{3}$$

The obtained data indicated that the stabilizer concentration (1%, 2%, 3% and 5%) had a significant synergistic effect on EE% with *p*-values of 0.0111, while stabilizer concentration had a significant antagonistic influence on particle size and zeta potential, with *p*-values of 0.001 and 0.0002, respectively. Meanwhile, the type of liquid lipid (oleic acid, Labrasol^®^, Capryol^®^ 90, and Miglyol^®^ 840) had a significant synergistic effect on particle size, with a *p*-value of 0.0152 and a significant antagonistic effect on EE% and PDI, with a *p*-value of <0.0001 and 0.001, respectively. The interaction effect of AB influenced zeta potential and PDI antagonistically with a *p*-value of 0.0025 and 0.045, respectively, and affected EE% synergistically, with a *p*-value of <0.0001.

**Figure 2:**
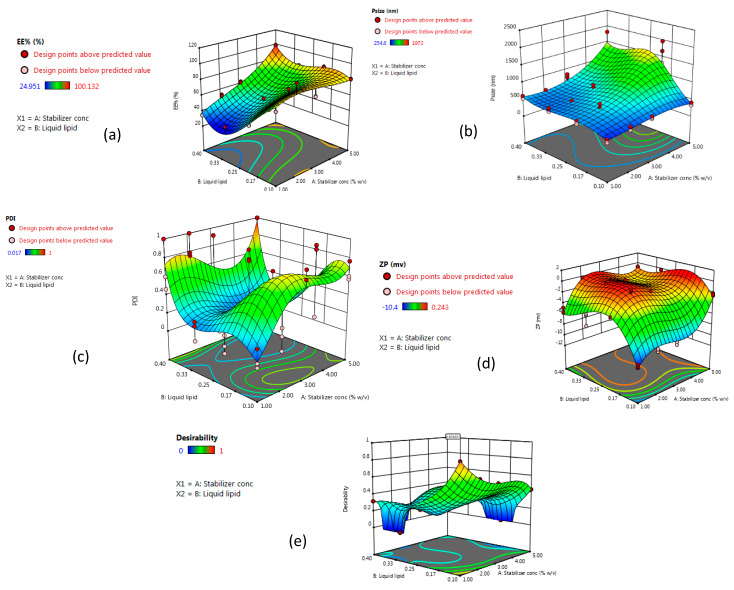
Three-dimensional response surface plots, showing the effect of stabilizer concentration (A) and liquid lipid type (B) on (**a**) EE%, (**b**) particle size, (**c**) polydispersity index (PDI), (**d**) zeta potential, and (**e**) desirability of the prepared nanostructured lipid carrier (NLC) formulations.

**Figure 3:**
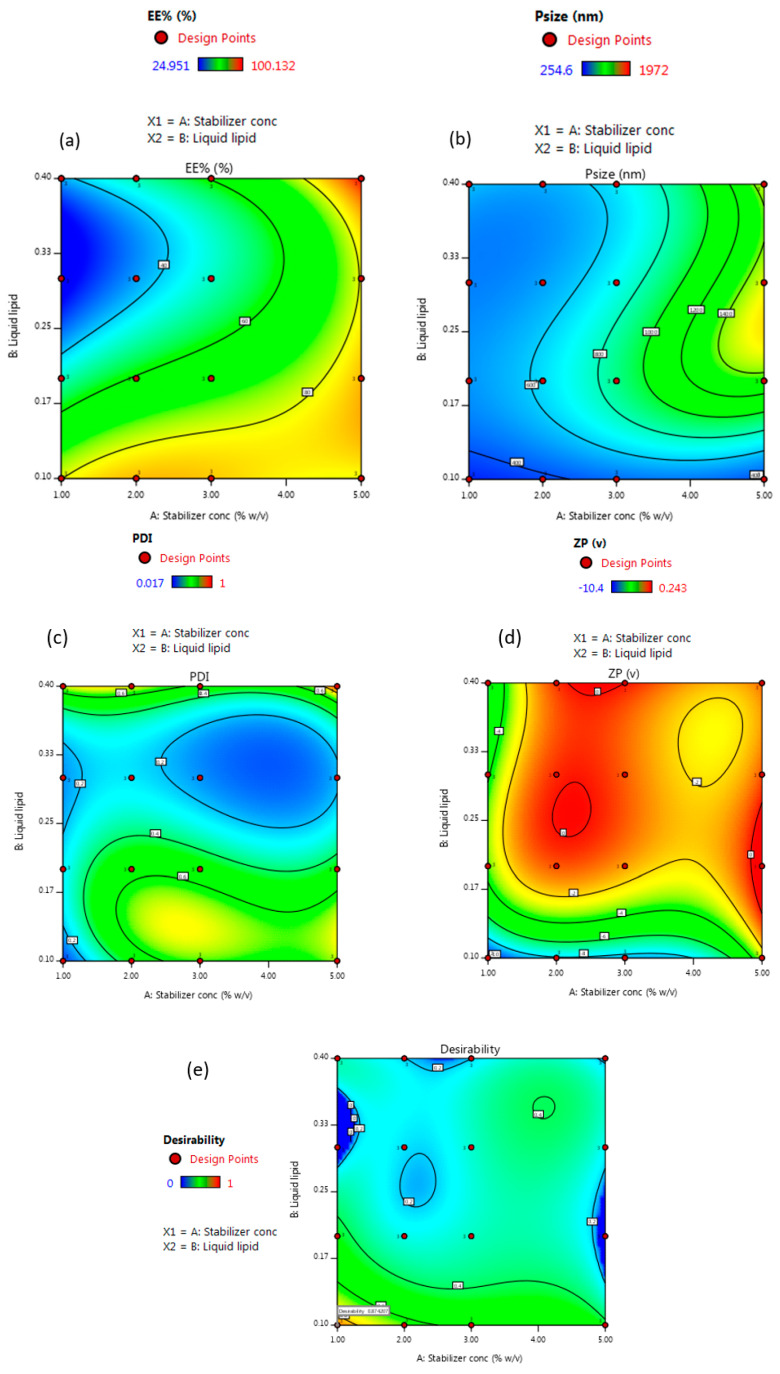
Two-dimensional contour plots showing the effect of stabilizer concentration (A) and liquid lipid type (B) on (**a**) EE%, (**b**) particle size, (**c**) PDI, (**d**) zeta potential, and (**e**) desirability of the prepared NLC formulations.

For evaluating responses values and coordinates, the data provided by Design-Expert^®^ software in the form of 3D response surface plots and 2D contour plots are displayed in Figure 2 and Figure 3. The observed responses of the experimental runs and the analysis of variance of a calculated model for a measured response such as *p*-value, F-ratio, and degrees of freedom are summarized in Table 2 and Table 3.

#### 3.1.1. Effect of Stabilizer Concentration on Particle Size

Poloxamer^®^ 407 was selected as a stabilizer to prepare NLC formulations. Poloxamer^®^ 407 is a hydrophilic nonionic surfactant that is composed of block copolymers of polyethylene oxide (PEO) and polypropylene oxide (PPO), with a hydrophilic-lipophilic balance (HLB) value of 22. The stabilizing effect comes from the ability of the hydrophobic PPO chains to be adsorbed on the particle surface, while the hydrophilic PEO chains withdrew from the surface to the aqueous medium, generating a stabilizer layer surrounding the particle [38].

The Z-average of the formed NLC formulations differed in the range of 306.6 ± 49.6 to 1658.7 ± 325.1, (Table 2). The concentration of the stabilizer has a significant antagonistic impact on the particle size of the developed NLCs (Figure 2 and Figure 3, Table 3), as a higher amount of stabilizer would readily emulsify the total lipids incorporated in the preparation, resulting in the conversion of the large-sized lipid particles into smaller ones. On the contrary, the insufficient amount of a stabilizer in lipid nanocarriers contributes to the instability and re-agglomeration of the particles [39].

Similar findings were previously reported by Rajinikanth and Chellian [40] during the preparation of 5-fluorouracil loaded nanostructured lipid carriers based hydrogel system using Poloxamer 188 and Solutol^®^ HS15 as stabilizers. The results confirmed the antagonistic effect between stabilizer concentration and particle diameter. Liu et al. [41] also noted the same effect upon encapsulation of isotretinoin-loaded SLNs, using tween 80 as a stabilizer. The results revealed that when tween 80 concentration increased, the particle size decreased accordingly. Besides, Prabhjot Kaur et al., indicated that the most influenced factor on the diameter of paclitaxel loaded NLC preparation was the surfactant concentration [42]. Particles with a diameter of less than 400 nm are preferred to improve the oral bioavailability of the therapeutics [43,44]. Therefore, the optimized formulation with optimum particle size will be selected.

#### 3.1.2. Effect of Stabilizer Concentration on Encapsulation Efficiency Percent (EE%)

Encapsulation efficiency (EE%) provides information on the amount of temazepam that can be incorporated into the lipid matrix of NLCs for better drug inclusion, protection, increased circulation time, and controlled release. EE% of the formed NLC formulations varied from 25.1 ± 0.1% to 99.8 ± 0.3% (Table 2). Increasing stabilizer concentration showed a significant synergistic influence on EE% of the formulated NLCs (Figure 2 and Figure 3, Table 3). The improved drug encapsulation within NLCs prepared using higher surfactant concentration resulted from the ability of surfactant to enhance the solubilization of the drug molecules within the lipid matrix of NLCs in addition to the stabilization of the formed particles [45]. Besides, increasing the surfactant concentration would promote the drug entrapment within the outer surface coating of NLCs. Similarly, Das et al. [46] found that varying the concentration of surfactant from 0.5% *w*/*w* to 3% *w*/*w*, resulted in increasing tretinoin EE% from 64.1 ± 4.2% to 80.8 ± 4.4%.

#### 3.1.3. Effect of Stabilizer Concentration on Zeta Potential

The surface properties of nanostructured lipid carriers play a vital role in affecting their interaction with the local environment, their solubility, stability, and clearance from the body [47].

The prepared NLC formulations were negatively charged, varying from (−0.08 ± 0.05 to −10.2 ± 0.3), (Table 2). The negative zeta potential values resulted from the ionization of glyceryl behenate, a fatty acid composing Compritol^®^ 888 ATO. Stabilizer concentration showed a significant antagonistic influence on surface charge values (Figure 2 and Figure 3, Table 3). The absolute value of zeta potential is affected by the nature of the stabilizer. As indicated, nonionic stabilizers such as Poloxamer^®^ 407 promote the steric stabilization of the NLCs by coating their surface, thus decreasing the electrostatic repulsion between the particles [48]. The combined effect of electrostatic and steric stabilization assists in the cellular uptake and minimizes the nonspecific interactions. Increasing the value of surface charge (either negative or positive) leads to higher macrophage scavenges, and clearance by the reticuloendothelial system. Consequently, this reduces the diffusion of nanocarriers to the target cells [49].

#### 3.1.4. Effect of Type of Liquid Lipid

The incorporation of liquid lipids into solid lipids is known to cause massive disorders in the crystal lattice [50]. Various liquid lipids were selected for the preparation of nanostructured lipid carriers, based on their safety profiles and biodegradability. The type of liquid lipid used for NLC formulations is known to influence the physicochemical characteristics of the formed particles; for instance, their hydrodynamic diameter, drug entrapment, viscosity, and subsequently predict their final fate [51].

Previous studies demonstrated the influence of varying the used liquid lipids on the physicochemical properties of the formed NLC preparations. For instance, Chinsriwongkul et al. [52] reported that using different liquid lipids (oleic acid, soybean oil, or medium-chain triglyceride) for the preparation of NLCs led to variable drug loading efficiency for all-trans retinoic acid. The maximum drug loading efficiency was obtained using oleic acid as a liquid lipid. Additionally, Shen et al. [53] tested the efficacy of Precifac^®^ ATO 5 and Miglyol^®^ 840, in forming NLC formulation for enhanced ocular delivery. The authors explained that increasing Miglyol^®^ 840 resulted in significant changes in the physicochemical properties and cellular uptake of the fabricated NLCs. The effect of liquid lipid type on the physicochemical properties of NLCs is best explained based on the combined effect of any of the viscosity of the oil used, its characteristic HLB value, miscibility between the solid and liquid lipid and the solubility of the drug in the used lipids. The physicochemical properties of liquid lipids used in the preparation of NLCs are shown in Table 4.

#### 3.1.5. Effect of Type of Liquid Lipid on Particle Size

Type of liquid lipid (oleic acid, Labrasol^®^, Capryol^®^ 90, and Miglyol^®^ 840) had a significant synergistic effect on particle size (Figure 2 and Figure 3, Table 3). As shown in Table 2, the particle size of NLCs prepared using oleic acid as liquid lipid was the lowest compared to other liquid lipids used. The higher viscosity of oleic acid (40 mPa·s, 20 °C), compared to Capryol^®^ 90 and Miglyol^®^ 840, resulted in the small particle size of the prepared NLCs. However, Labrasol^®^, a nonionic oil-in-water surfactant, although having a higher viscosity than oleic acid (80–110 mPa·s, 20 °C), showed a larger particle size. This could be explained based on the higher tendencies of Labrasol^®^ for interaction with poloxamer^®^ 407, leading to the formation of micelles at higher concentrations [54]. The impact of a combination of oleic acid and compritol^®^888 ATO on the particle size of NLCs was previously reported. Oleic acid is known to reduce the viscosity and surface tension of the formed nanosystem when mixed with Compritol^®^ 888 ATO and thus leads to the production of NLCs of small-sized particles [55,56].

#### 3.1.6. Effect of Type of Liquid Lipid on Zeta Potential

The interaction effect of AB affected zeta potential antagonistically (Figure 2 and Figure 3, Table 3). Extravagant ionization of numerous carboxylic groups present in oleic acid composition led to an increase in negative charge and ZP of the developed NLCs [57]. Besides its effect on the stability of the formed particles, zeta potential is an important parameter that affects the passage of nanoparticles through the BBB. The surface charge must also be considered for the prediction of toxicity and brain distribution profiles. Neutral and low anionic nanocarriers can be safely used as colloidal drug carriers targeting the brain, as they do not have an acute effect on BBB integrity. On the contrary, high concentrations of anionic and cationic nanocarriers have an immediate toxic disruptive effect on BBB. As a result, most of the nano-formulations used in brain delivery have moderate ZP values (between −1 to −15 mV) [58,59,60]. Improving the delivery of NLCs to brain cells requires systems that target and cross BBB more efficiently, and are also slowly removed from the bloodstream. Thus, for the selection of optimum NLC formulation, the small negative surface charge is expected to be suitable for overcoming the energy barrier of electrical repulsion associated with the negatively charged mucus gel layer covering the enterocyte liner and thus enable the effective absorption of these nano colloids in vivo.

#### 3.1.7. Effect of Type of Liquid Lipid on Encapsulation Efficiency (EE%)

The incorporation of oleic acid as a liquid lipid resulted in a significant increase in the entrapment efficiency of temazepam compared to other liquid lipids used (Figure 2 and Figure 3, Table 3). This finding can be explained based on the higher affinity of lipophilic drugs, such as temazepam, to the less polar and more lipophilic oil phase, resulting in better drug miscibility. Oleic acid with a low HLB value (HLB = 1), has a lipophilic nature that allows better loading of lipophilic temazepam.

On the contrary to the obtained results, Rania A. Sanad et al. [61] indicated the superior oxybenzone entrapment efficiency within NLC prepared using Miglyol^®^ 812 liquid lipid, when compared to oleic acid. Although the solid lipid used in their study was different (Glyceryl monostearate), the authors explained their finding based on the composition of Miglyol^®^ 812, which is composed of a mixture of triglycerides able to constitute imperfect crystals that offer sufficient space to include more drugs [62], compared with oleic acid, that is made of a monounsaturated fatty acid [63]. Additionally, Shady Ali Swidan et al. [64] revealed that the use of Capryol^®^ 90 as a liquid lipid resulted in the enhancement of paclitaxel encapsulation percentage, in comparison with the use of oleic acid. The result was explained based on the structural difference between Capryol^®^ 90 and the solid lipid used (Glyceryl monostearate), which led to the formation of a disordered system able to include higher drug amounts [63].

#### 3.1.8. Effect of Type of Liquid Lipid on PDI

The accumulation of lipid nano-carriers in the target tissue is strongly dependent on their physicochemical properties, including particle size distribution. Therefore, the successful formulation of efficient NLCs necessitates the preparation of homogenous NLC dispersions of a particular size [65]. The polydispersity index (PDI) is a parameter used to estimate the uniformity of nanodispersions. Moreover, PDI can evaluate the tendency for aggregation and the stability of the formed nanodispersions [66]. The numerical value of PDI ranges from 0.0 (for uniform monodisperse particles) to 1.0 (for highly polydisperse particles with varied particle size populations). The outcome of increasing surfactant concentration and varying liquid lipids on PDI values is presented in Table 2. In drug delivery applications, a PDI of 0.3 and below is considered to be acceptable and indicates a homogenous distribution of lipid nanoparticles [67]. High PDI values for some NLC batches indicate relatively broad size distribution. During the initial formulation phase, the heat applied for the formation of emulsion leads to the melting of solid lipid, however, during the cooling down process, the miscibility of the oil in the solid lipid decreased, causing phase separation before achieving the desired particle size [68,69]. The particle size in emulsions is strongly correlated to the viscosity of the dispersed phase and the interfacial tension between the dispersed and continuous phases [70]. Therefore, the introduction of high melting point lipids as a dispersed phase contributed to raising the viscosity ratio between the dispersed and continuous phases, resulting in NLCs with larger particle size and higher PDI values [71]. Consequently, the use of a higher concentration of surfactant in the preparation of the pre-emulsion would aid in reducing the particle size and PDI values.

#### 3.1.9. Selection of Optimized NLC Formulation

According to the developed polynomial Equations (1)–(4), optimization was done using the desirability approach, in order to obtain the levels of A and B that will minimize particle size, PDI, and zeta potential, while maximizing EE% for imperative brain targeting efficiency. The desirability value ranges from (0–1). A formula is considered acceptable when the desirability value approaches 1. Therefore, the formulation that had the highest desirability value was selected. The predicted stabilizer concentration (A) chosen by the software was 1% *w*/*v*, while the type of selected liquid lipid (B) was oleic acid. To validate the values of the responses, the responses obtained from the optimized formulation were compared to the responses predicted by the software. The experimental values for responses of the optimum formula are in agreement with the predicted values produced by the software confirming the validity of the design.

The optimized formulation composes of Compritol^®^ 888 ATO: 75 mg, oleic acid: 25 mg in 2 mL of acetone/ethanol (3:1 *v*/*v*) and Poloxamer^®^ 407: 0.3 g in 30 mL distilled water, exhibited particle size of 306.6 ± 49.6 nm, encapsulation efficiency (EE%) of 75.2 ± 0.1%, zeta potential of −10.2 ± 0.3 mV and PDI of 0.09 ± 0.10 (Table 2).

### 3.2. Characterization of Temazepam-Loaded NLC Formulation

#### 3.2.1. FTIR Analysis

**Figure 4:**
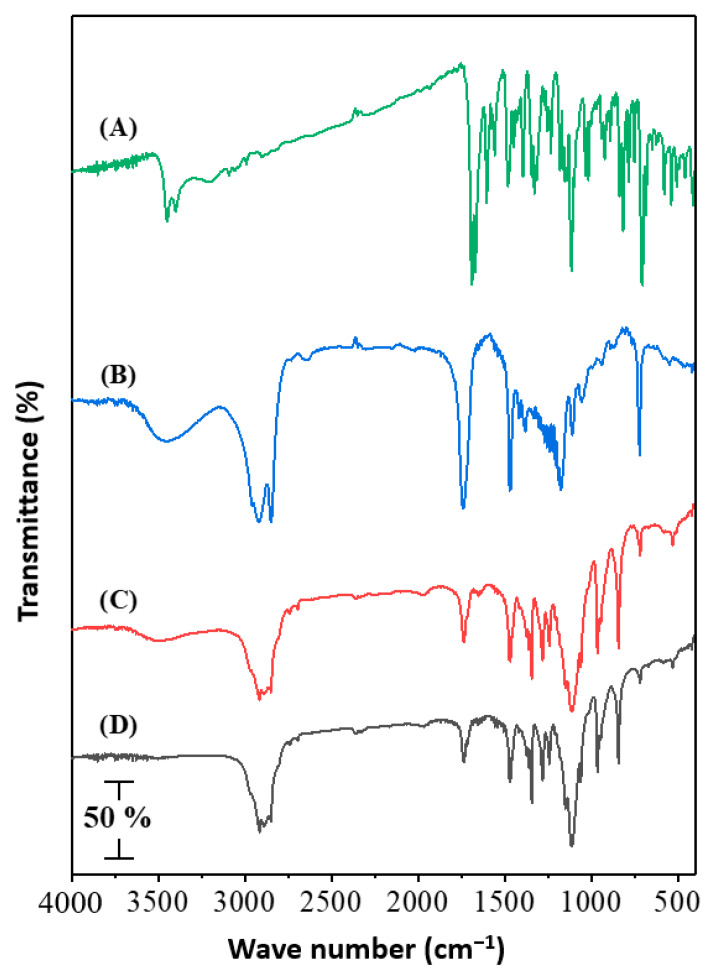
FTIR spectra of (**A**); Temazepam powder, (**B**) Compritol^®^ 888 ATO powder, (**C**) Plain NLC-1 freeze-dried powder, and (**D**) Temazepam NLC-1 freeze-dried powder.

FTIR analysis was conducted in the range of 4000–400 cm^−1^ to detect any chemical or physical interaction between temazepam and the lipid matrix (Figure 4, Panel A−D). The spectrum of pure temazepam (Figure 4A) showed typical absorption peaks at 648 cm^−1^, 1115 cm^−1^_,_ and 1670–1691 cm^−1^ (Fermi doublet), corresponding to C–Cl, C–OH, and C=O, respectively, consistent with the reported values [16]. The main absorption peaks of Compritol^®^ 888 ATO (Figure 4B) were observed at 3300 cm^−1^, 2815 cm^−1^_,_ and 1738 cm^−1^ for O–H, C–H, and C=O stretching, respectively [72]. The FTIR spectra of the plain NLC-1 and the temazepam NLC-1 preparations were almost the same (Figure 4C,D). The disappearance of distinctive temazepam peaks in the temazepam NLC-1 spectrum (Figure 4D) confirmed the successful incorporation of temazepam into the lipid matrix [73]. The entrapment of temazepam in NLCs depends on the dissolution of the drug in lipids. Similar findings have previously been reported [74,75].

#### 3.2.2. DSC Analysis

**Figure 5:**
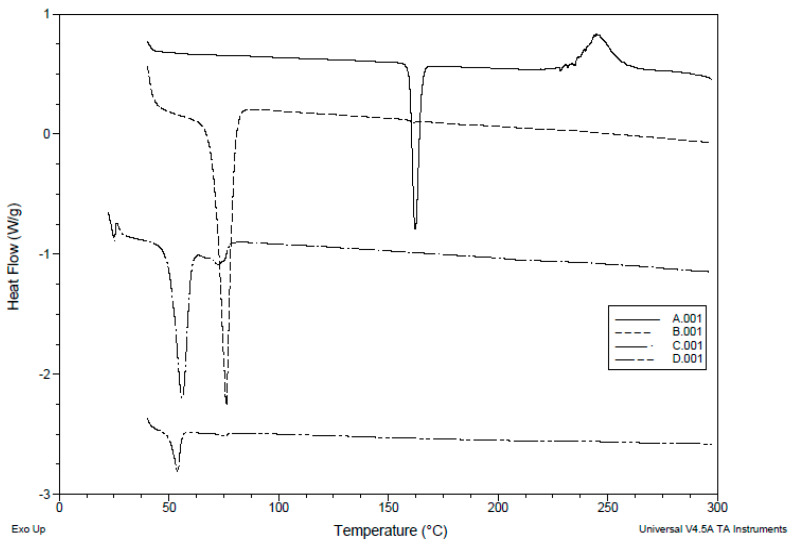
DSC thermogram of (A); Temazepam powder, (B) Compritol^®^ 888 ATO powder, (C) Plain NLC-1 freeze-dried powder, and (D) Temazepam NLC-1 freeze-dried powder.

DSC analysis adds a great value in evaluating drug-lipid interactions and mixture behaviors of Compritol^®^ 888 ATO and oleic acid. DSC thermograms of temazepam and Compritol^®^ 888 ATO are presented in Figure 5, Panel A and B, showing sharp endothermic peaks at 160.71 °C and 72.31 °C, respectively, associated with their melting points. On the other hand, a noticeable reduced endothermic peak of Compritol^®^ 888 ATO was recorded in the thermogram of freeze-dried plain NLC formulation and freeze-dried temazepam NLC-1 formulation (Figure 5, Panel C and D), compared with that of pure Compritol^®^ 888 ATO, presumably due to the colloidal size range of the lipid particles. Moreover, the shift in the melting point observed in the thermogram of NLC preparations may be a consequence of the interaction of Compritol^®^ 888 ATO with oleic acid during the preparation process [76]. The disappearance of the temazepam peak at 160.71 °C in the thermogram of the NLC-1 formulation indicates the solubilization of temazepam in the lipid phase and the existence of the drug in either a molecularly dispersed or amorphous state upon incorporation into NLC matrix [77], Figure 5 (Panel D). A less ordered crystal or amorphous lipid matrix resulting from the interaction between solid–lipid and liquid–lipid is favored for incorporating higher drug content [72].

#### 3.2.3. X-ray Diffraction

**Figure 6:**
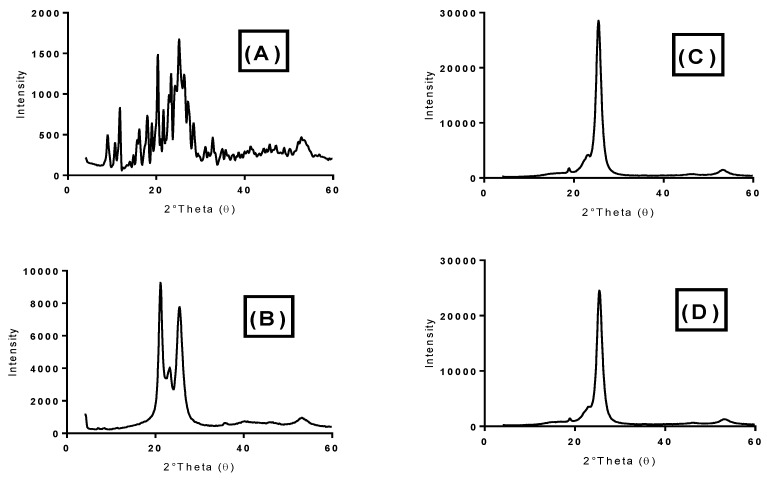
X-ray diffraction patterns of (**A**) Temazepam powder, (**B**) Compritol^®^ 888 ATO, (**C**) Plain NLC-1 freeze-dried powder, and (**D**) Temazepam NLC-1 freeze-dried powder.

The XRD patterns of temazepam, Compritol^®^ 888 ATO, plain NLC freeze-dried powder, and freeze-dried temazepam NLC-1 formulation are shown in Figure 6A–D. The XRD pattern of temazepam showed numerous characteristic diffraction peaks at 2*θ* of 20.37°, 23.37°, and 25.18°, confirming its crystalline nature (Figure 6A) [16]. Such characteristic sharp peaks of temazepam were absent in the diffraction patterns of the freeze-dried temazepam NLC-1 formulation (Figure 6D), which ensured the loss of crystallinity and the presence of the drug in an amorphous state within the NLC lipid matrix [77]. The results of the XRD pattern are consistent with the DSC data. A similar finding was reported by Khan et al., where reduced crystallinity was observed for lopinavir when loaded into nanostructured lipid carriers due to improved drug solubility in glyceryl behenate, a fatty acid composing Compritol^®^ 888 ATO [78]. The sharp characteristic peaks of Compritol^®^ 888 ATO at 21.16° and 25.48° (Figure 6B) [79] were reduced in the plain, as well as the temazepam loaded NLC preparation (Figure 6C,D), due to the polymorphic crystalline transitions of Compritol^®^ 888 ATO lipid upon heating [80,81].

Overall, the efficient loading of temazepam within the lipid matrix of NLC was evidenced by the loss of drug crystallinity confirmed by the absence of its characteristic peaks in FTIR spectra, DSC thermograms, and XRD patterns.

#### 3.2.4. Morphological Analysis

**Figure 7:**
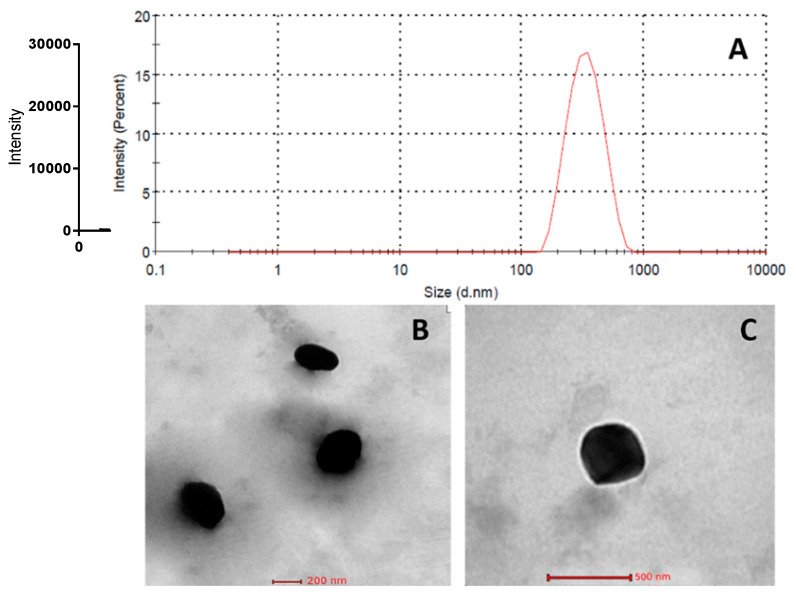
Representative intensity-weighted particle size distribution profile of temazepam NLC-1 suspension (**A**); and TEM micrographs (×52,000) (**B**) and (×150,000) (**C**).

The TEM images of the optimized temazepam NLC-1 formulation are shown in Figure 7, Panels B and C. The prepared NLC formulation showed a smooth mono-dispersed spherical structure with a characteristic border between each particle. The particle diameter determined by TEM analysis is consistent with that obtained by DLS measurements (Figure 7A).

#### 3.2.5. In Vitro Release Profile of Temazepam Loaded NLC Formulation

**Figure 8:**
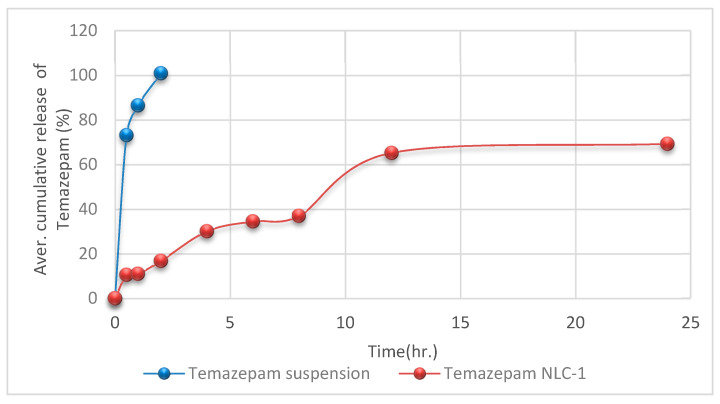
In vitro release profile of temazepam NLC-1 formulation compared to temazepam suspension in phosphate buffer saline (PBS, pH 7.4) at 37 °C. Data are expressed as mean ± S.D. (*n* = 3).

An in vitro release study of temazepam from the optimized NLC-1 formulation was carried out in PBS, pH 7.4 at 37 °C, using a modified dialysis membrane diffusion technique. The in vitro release profile of temazepam NLC-1 was compared with that of temazepam suspension (Figure 8). Considering the poor aqueous solubility of temazepam (53.4 µg/mL) [82], sink conditions were maintained using 40 mL PBS, pH 7.4 release vehicle for the total drug content of the NLC formulation or drug suspension (141 µg), so it seems highly unlikely that the release of the drug would be restricted by exceeding the sink conditions.

Temazepam suspension demonstrated maximum drug release (100.9 ± 1.0%) in 2 h due to its lipophilic nature, confirming the permeability of temazepam through the dialysis membrane used. The temazepam NLC-1 formulation showed a biphasic drug release profile with an initial rapid drug release of 30.1 ± 0.6% in the first 4 h, followed by a more sustained drug release (69.3 ± 0.3%) up to 24 h. The best-fit model for the temazepam NLC-1 in vitro release profile was the Higuchi diffusion model, with correlation coefficient (*r*^2^) 0.9724.

The biphasic release pattern of the temazepam NLC-1 preparation could be explained by the partitioning of the drug between the aqueous and lipid phases during the NLC formulation. During development, the high temperature and the presence of a stabilizer lead to increased drug solubility in the aqueous phase. But, during the cooling step, the drug would disperse again in the lipid layer. The high melting point of Compritol^®^ 888 ATO quickly solidifies the enriched solid lipid core layer. As a result, the deposition of the outer shell of a liquid lipid would follow. The drug on the outer oleic acid shell layer contributes to its burst release, followed by a controlled and sustained release of the drug included in the solid lipid matrix.

These findings were consistent with previous literature reports that explained such a biphasic drug release pattern for NLC-encapsulated drug molecules [28,77,83]. The sustained released drug-loaded NLC formulation is predicted to enhance drug residences within the brain, thus eliminating the adverse effects accompanying repeated drug administrations. Correlation of these results with the pharmacokinetics in vivo performance evaluation of this anxiolytic drug in a relevant animal model is necessary to assess the therapeutic possibilities of this novel oral delivery system for temazepam.

### 3.3. In Vivo Studies

The management of neurological disorders represents the main challenge for pharmaceutical companies. Recently, various strategies based on nano-platforms have been developed to enhance the brain delivery of therapeutics across BBB [84], despite the lipophilic nature of temazepam that allows it to pass through BBB. However, it is quickly spread outside the brain, resulting in drug accumulation and adverse complications [36]. Successful inclusion of temazepam in the NLC matrix is predicted to enhance stability and bioavailability during gastrointestinal transit. Besides, the drug has the ability to penetrate the BBB.

#### 3.3.1. Gamma Scintigraphic Imaging

**Figure 9:**
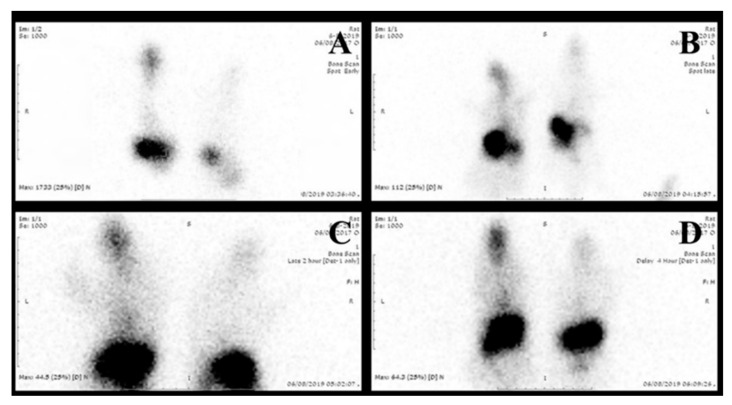
Serial anterior planar images of two rats at (**A**) 30 min, (**B**) 1 h, (**C**) 2 h, and (**D**) 4 h. The right one received 77.5 µCi of the oral ^Tc-99m^ labeled temazepam NLC-1 formulation; the left one received 77.5 µCi of the ^Tc-99m^ labeled temazepam oral suspension. All of the images showed higher uptake of the brain of the right one.

**Figure 10:**
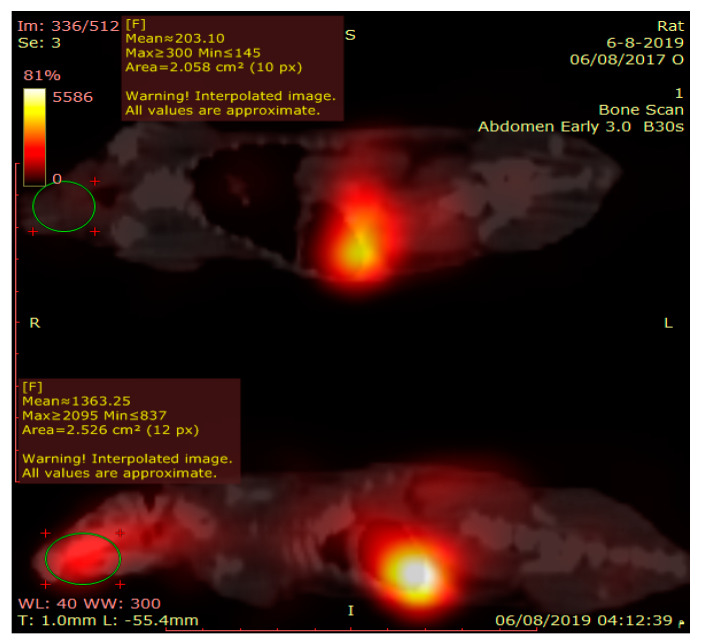
Sagittal view of fused SPECT/CT image of two rats, acquired after 30 min of drug administration. The upper one received ^Tc-99m^ labeled temazepam oral suspension; the lower one received ^Tc-99m^ labeled temazepam NLC-1 formulation. The region of interest (ROI) drawn on the brain shows higher counts in the lower one.

**Figure 11:**
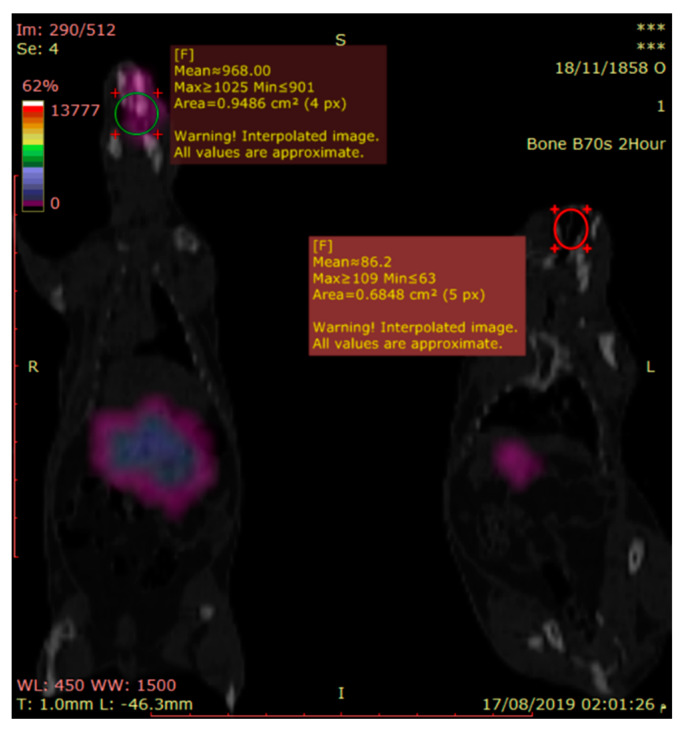
Sagittal view of fused SPECT/CT image of two rats, acquired after two hours of drug administration. The right one received 77.5 µCi of the oral ^Tc-99m^ labeled temazepam NLC-1 formulation; the left one received the same dose of the ^Tc-99m^ labeled temazepam oral suspension. Evidence of higher brain radioactivity is shown in the right one.

Gamma scintigraphy was performed to compare the potential of orally administered ^99m^Tc-temazepam suspension and ^99m^Tc-temazepam NLC-1 formulation to cross BBB. The gamma scintigraphic images give a clear picture of the exact location of the radiolabeled formulation. The images showed that both the radiolabeled drug suspension and the NLC formulation were able to cross BBB and locate within the brain. However, the photos clearly showed that ^99m^Tc-temazepam NLC-1 formulation was able to localize within the brain at higher concentrations, compared to ^99m^Tc-temazepam suspension at all the studied time points. This observation was confirmed by the higher mean counts in the images (Figure 9, Figure 10 and Figure 11).

#### 3.3.2. Pharmacokinetics and Brain Distribution

The biodistribution of ^99m^Tc-temazepam following peroral administration of ^99m^Tc-temazepam suspension and ^99m^Tc-temazepam NLC-1 preparation to rats was monitored, and the radioactivity was determined at predefined time points up to 6 h. The outcomes were presented as a percentage of the administered dose. The brain/blood ratio of temazepam for both groups at all the studied time intervals was also estimated and displayed in Table 5.

The ratio of brain/blood for the drug was observed to be higher following the administration of ^99m^Tc-temazepam NLC-1 formulation at all-time intervals. The drug concentration within the brain after oral administration of ^99m^Tc-temazepam NLC-1 preparation was determined to be significantly higher (*p* < 0.05) at all sampling time intervals, in comparison to that after oral administration of ^99m^Tc-temazepam suspension. The calculated values of the brain/blood ratio demonstrated that radiolabeled NLC-1 formulation kept its level steadily higher, in comparison to a drug suspension, indicating the superior ability of NLC formulation to achieve a sustained level of drug in blood and brain when compared to drug suspension throughout the whole study duration (6 h).

These conclusions are consistent with the findings obtained from Ghazizadeh and co-workers, who compared the biodistribution of free ^99m^Tc-isoniazid and ^99m^Tc-isoniazid loaded solid lipid nanoparticles. The obtained results revealed the superior drug retention in all body organs after the oral administration of drug-loaded solid lipid nanoparticles compared to the free drug [85]. Besides, the successful itraconazole transport to the brain after its encapsulation within a nanostructured lipid carrier formulation was reported by Lim and colleagues. The concentration of the drug which reached the brain was determined to be two folds higher than the free unloaded drug [28].

**Figure 12:**
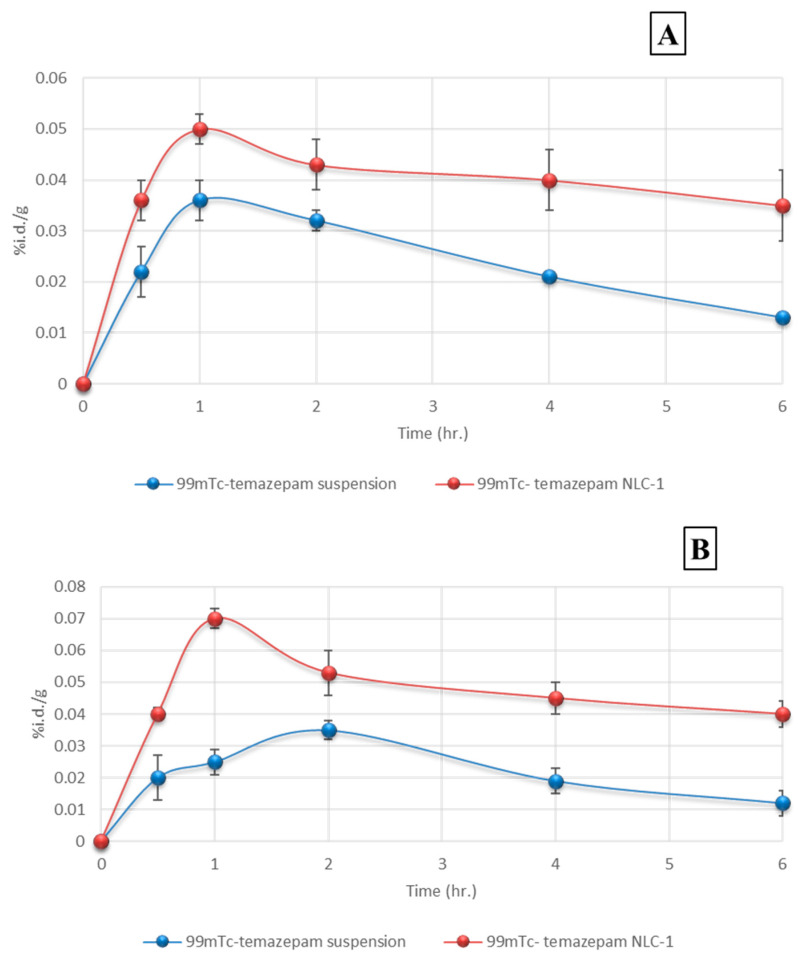
Concentration–time profiles of ^99m^Tc-temazepam suspension and ^99m^Tc-temazepam NLC-1 formulation after oral administration to rats. Panel (**A**) Plasma; and panel (**B**) brain. Each data point represents the mean ± SD of six determinations.

Figure 12A depicts the plasma concentration-time profile of ^99m^Tc-temazepam following peroral administration of ^99m^Tc-temazepam NLC-1 formulation compared to ^99m^Tc-temazepam suspension. The pharmacokinetic data are summarized in Table 6.

Relative rate of uptake (Re)=(AUC brain) Tem−NLC formulation(AUC brain) Tem−suspension

$$\mathrm{Ratio}\;\mathrm{of}\;\mathrm{Peak}\;\mathrm{concentration}\;\left(\mathrm{Ce}\right)=\frac{(C_{\max\mathrm{brain}})\;\mathrm{Tem}-\mathrm{NLC}\;\mathrm{formulation}}{(C_{\max\mathrm{brain}})\;\mathrm{Tem}-\mathrm{suspension}}$$

Drug targeting index (DTI)=(AUC brainAUC plasma) Tem−NLC formulation(AUC brainAUC plasma) Tem−suspension

The area under concentration (AUC_0–∞_) determined following the peroral administration of ^99m^Tc-temazepam NLC-1 formulation (0.68 ± 0.25%id/g*h) was significantly higher compared with that determined after oral administration of ^99m^Tc-temazepam suspension (0.23 ± 0.11%id/g*h), *p* < 0.05. Besides, the relative bioavailability was elevated (292.7%) and the *C*_max_ value was higher (0.057 ± 0.012% id/g). These observations confirm the significant enhancement in the oral absorption of ^99m^Tc-temazepam from NLC-1 formulation compared to the free drug suspension. Enhanced *t*_1/2_, extended MRT _(0–∞)_, and reduced CL estimated for ^99m^Tc-temazepam NLC-1 administered rats confirm longer drug residence time and delayed elimination. The time to attain maximum drug concentration (*T*_max_) did not differ between both groups. These results depict that ^99m^Tc-temazepam NLCs could prolong the blood circulation of temazepam and improve therapeutic efficacy through the raised AUC_0–∞_.

A significant improvement in relative bioavailability following oral administration of the drug-loaded NLC-1 formulation could be explained based on; the small particle size and large surface area of NLCs that facilitate better contact with gastrointestinal mucosa and improve the rate of drug absorption [86]. The presence of a surfactant in NLC formulation could also inhibit the P-glycoprotein multidrug efflux system in the lower gastrointestinal tract [87,88]. Moreover, the NLC system is thought to enhance intestinal cellular uptake and lymphatic transport and hence leads to augmented temazepam absorption [78,89]. Additionally, small nano-sized particles transported in blood circulation are difficult to be taken up by phagocytosis [90].

The concentration-time profile of ^99m^Tc-temazepam in brain tissues of rats after oral administration of ^99m^Tc-temazepam NLC-1 formulation and the ^99m^Tc-temazepam suspension is shown in Figure 12B. Pharmacokinetic data and targeting efficiency were calculated (see Table 6). Following peroral administration, the maximum drug concentration (*C*_max_) found in the brain was markedly increased in the group receiving ^99m^Tc-temazepam NLC-1 formulation (0.070 ± 0.001% id/g), compared to the control group receiving ^99m^Tc-temazepam suspension (0.033 ± 0.006% id/g).

Furthermore, a 3.4-fold higher (AUC_0–∞_) value was observed for the ^99m^Tc-temazepam NLC-1 receiving group compared to the control group. In addition to; longer *t*_1/2_ and MRT _(0–∞)_ values and shorter *T*_max_. All these observations signify the slower clearance, prolonged retention, and the facilitated brain uptake of ^99m^Tc-temazepam, following the oral administration of NLC-1 formulation.

For the better estimation of blood-to-brain direct transport and targeting efficiency, drug targeting index (DTI), the peak concentration ratio (Ce), and the relative uptake rate (Re) were calculated. DTI equals (1.16), which suggests a preferentially targeted brain distribution for optimum NLC-1 formulation compared to oral temazepam suspension. Besides, Ce = 2.12 and Re of 3.38, assuring a dramatic augmentation of temazepam delivery within the brain (Table 6).

Several strategies could be postulated for the improved distribution of temazepam through BBB via NLC formulation. The small hydrodynamic diameter and lipophilic nature of NLC formulation enable better contact with BBB and enhanced permeation via the concentration gradient effect [91]. The presence of surfactant in NLC formulation could contribute to the enhanced membrane fluidity, stopping brain endothelial cell efflux pumps, as well as better attachment to lipoprotein receptors that support brain delivery [28]. Besides, NLCs transport across BBB could be mediated via endocytosis, transcytosis, or paracellular opening of tight junctions [1,92]. The combination of these mechanisms might lead to the enhanced brain uptake of temazepam-NLCs. Further studies can be carried out to delineate the exact mechanisms that contribute to the enhanced temazepam-NLC brain uptake via using in vitro BBB models. The possible pathways of cellular uptake for the particles can be investigated by pre-incubation of the cells with specific inhibitors of endocytosis, followed by incubation with NLCs.

Based on the aforementioned experimental findings, the optimized temazepam loaded NLC formulation could augment the intestinal absorption and oral bioavailability, in addition to improved blood to brain targeting affinity.

## 4. Conclusions

In this study, temazepam-loaded nanostructured lipid carriers (NLCs) were successfully developed and optimized using a 4^2^ full factorial design. The optimized temazepam NLC-1 preparation demonstrated a high encapsulation efficiency (75.2 ± 0.1%), with negative zeta potential and nanometer particle size. Moreover, NLCs were able to sustain drug release compared to an unloaded drug suspension. Using a lipid carrier to encapsulate this hydrophobic benzodiazepine effectively translates into a significant enhancement in the relative drug bioavailability (292.7%), in addition to a 3.4-fold improvement in the relative rate of brain uptake when compared to temazepam suspension. The small particle size, the large surface area, and the lipophilic nature of temazepam NLCs appear to facilitate NLC transport to the brain via combined mechanisms (endocytosis, transcytosis, membrane fluidization, the opening of tight junctions, and/or the inhibition of efflux pumps). Consequently, the implementation of NLCs for mediating brain delivery is a promising strategy for the management of neurological disorders.

## 5. Recommendations and Future Perspectives

Future directions will focus on conjugating the developed temazepam loaded NLC formulation, with targeting ligands such as transferrin and lactoferrin, which have been applied recently as brain targeting moieties due to the overexpression of brain endothelial cells and glioma cells of BBB to these receptors. Consequently, these ligands can carry the NLCs through BBB, leading to more enhanced brain-specific drug delivery.

## Figures and Tables

**Table 1:** Levels of dependent and independent variables used in the factorial design.

Factors	Levels				Responses	Constrains		
						Minimum	Maximum	Goal
A	1	2	3	5	R_1_	24.95	100.13	Maximize
					R_2_	254.6	1972.0	Minimize
B	Oleic	Labrasol^®^	Capryol^®^ 90	Miglyol^®^ 840	R_3_	0.017	1.00	Minimize
					R_4_	−10.40	0.24	Minimize

**Table 2:** Experimental runs and their observed responses.

Run	Stabilizer Conc. (%*w*/*v*)	Liquid Lipid Type	EE (%) (±SD)	ɀ Average d.nm (±SD)	PDI (±SD)	Zeta Potential (ZP) mV (±SD)
1	1	oleic	75.2 ± 0.1	306.6 ± 49.6	0.09 ± 0.10	−10.2 ± 0.3
2	2	oleic	99.8 ± 0.3	401.6 ± 51.8	0.21 ± 0.12	−8.8 ± 0.3
3	3	oleic	75.6 ± 0.3	440.2 ± 36.8	0.76 ± 0.06	−8.8 ± 0.3
4	5	oleic	81.3 ± 0.4	357.9 ± 43.1	0.65 ± 0.10	−2.4 ± 0.2
5	1	Labrasol^®^	46.5 ± 0.1	477.8 ± 50.2	0.11 ± 0.09	−3.6 ± 0.1
6	2	Labrasol^®^	56.5 ± 0.1	656.7 ± 105.8	0.93 ± 0.06	0.06 ± 0.19
7	3	Labrasol^®^	73.9 ± 0.2	695.8 ± 163.5	0.34 ± 0.33	−1.1 ± 0.0
8	5	Labrasol^®^	86.9 ± 0.7	1658.7 ± 325.1	0.56 ± 0.46	−0.19 ± 0.07
9	1	Caproyl^®^ 90	31.6 ± 0.1	512.7 ± 56.7	0.14 ± 0.11	−5.2 ± 1.2
10	2	Caproyl^®^ 90	25.1 ± 0.1	489.2 ± 66.6	0.10 ± 0.09	−0.08 ± 0.05
11	3	Caproyl^®^ 90	50.8 ± 0.1	708.9 ± 90.1	0.25 ± 0.14	−0.31 ± 0.03
12	5	Caproyl^®^ 90	79.9 ± 0.4	1251.3 ± 228.8	0.09 ± 0.01	−0.21 ± 0.02
13	1	Miglyol^®^ 840	34.5 ± 0.3	558.5 ± 42.1	0.69 ± 0.28	−5.4 ± 0.5
14	2	Miglyol^®^ 840	52.0 ± 0.9	490.1 ± 76.8	0.85 ± 0.13	−0.64 ± 0.59
15	3	Miglyol^®^ 840	61.9 ± 0.8	740.2 ± 111.7	0.46 ± 0.45	−0.47 ± 0.06
16	5	Miglyol^®^ 840	98.4 ± 0.8	1421.7 ± 376.2	1 ± 0	−0.80 ± 0.11

**Table 3:** Analysis of variance of the final models for measured responses.

**Parameters**	**SS**	**DF**	**MS**	**F**	***p*-Value**	**Parameters**	**SS**	**DF**	**MS**	**F**	***p*-Value**
**Model**	**EE%**					**Model**	**PDI**				
Cubic	22266.26	9	2474.03	50.3	<0.0001	Cubic	2.36	9	0.26	5.04	0.0002
A	351.4	1	351.4	7.2	0.0111	A	0.06	1	0.06	1.21	0.2793
B	1981.7	1	1981.7	40.3	<0.0001	B	0.67	1	0.67	12.79	0.0010
AB	3294.4	1	3294.4	67.0	<0.0001	AB	0.22	1	0.22	4.30	0.0451
A^2^	12.2	1	12.2	0.25	0.6220	A^2^	0.02	1	0.02	0.44	0.5092
B^2^	2570.3	1	2570.3	52.3	<0.0001	B^2^	0.68	1	0.68	13.01	0.0009
A^2^B	217.1	1	217.1	4.4	0.0425	A^2^B	0.28	1	0.28	5.34	0.0266
AB^2^	493.6	1	493.6	10.0	0.0031	AB^2^	0.10	1	0.10	1.98	0.1673
A^3^	40.9	1	40.9	0.83	0.3678	A^3^	0.09	1	0.09	1.73	0.1971
B^3^	781.9	1	781.9	15.9	0.0003	B^3^	0.62	1	0.62	11.83	0.0015
**Parameters**	**SS**	**DF**	**MS**	**F**	***p*-Value**	**Parameters**	**SS**	**DF**	**MS**	**F**	***p*-Value**
**Model**	**Particle Size**					**Model**	**Zeta Potential**				
Cubic	3.6 × 10^−3^	9	4.0 × 10^−4^	30.1	<0.0001	Cubic	558.7	9	62.1	121.7	<0.0001
A	1.7 × 10^−4^	1	1.7 × 10^−4^	12.7	0.0010	A	9.1	1	9.1	17.8	0.0002
B	8.7 × 10^−5^	1	8.7 × 10^−5^	6.5	0.0152	B	0.67	1	0.67	1.3	0.2578
AB	1 × 10^−4^	1	1 × 10^−4^	7.35	0.0101	AB	5.4	1	5.4	10.5	0.0025
A^2^	2.9 × 10^−7^	1	2.9 × 10^−7^	0.02	0.8822	A^2^	5.3	1	5.3	10.5	0.0026
B^2^	6.6 × 10^−4^	1	6.6 × 10^−4^	49.1	<0.0001	B^2^	118.9	1	118.9	233.2	<0.0001
A^2^B	1.6 × 10^−4^	1	1.6 × 10^−4^	11.9	0.0014	A^2^B	35.9	1	35.9	70.4	<0.0001
AB^2^	1.2 × 10^−4^	1	1.2 × 10^−4^	8.8	0.0052	AB^2^	9.7	1	9.7	18.9	0.0001
A^3^	1.1 × 10^−5^	1	1.1 × 10^−5^	0.81	0.3726	A^3^	23.2	1	23.2	45.6	<0.0001
B^3^	2.4 × 10^−4^	1	2.4 × 10^−4^	17.6	0.0002	B^3^	25.2	1	25.2	49.3	<0.0001

**Table 4:** Physicochemical properties of liquid lipids used in the preparation of NLCs.

Liquid Lipid Type	General Class	Chemical Name	C-Length	M.wt Average (g/mol)	Viscosity (20 °C) (mPa·s)	Hydrophilic-Lipophilic Balance (HLB)
Oleic acid	Fatty acids	Octadec-9-enoic acid	C18	282.5	40	1
Labrasol^®^	Polyglycolyzed glycerides	Caprylo caproyl Polyoxyl-8 glycerides	C8–C10	400	80–110	12
Capryol^®^ 90	PG fatty acid esters	Propylene glycol monocaprylate (type II)	C8	202.29	20	5
Miglyol^®^ 840	PG fatty acid esters	Propylene glycol diester of saturated vegetable fatty acids	C8-C10	709	9–12	<10

**Table 5:** Distribution of temazepam in blood and brain at different sampling time points.

Formulation	Organ	Time Points (h)				
		0.5 h	1 h	2 h	4h	6 h
^99m^Tc-temazepam suspension	Blood	0.022 ± 0.005	0.036 ± 0.004	0.032 ± 0.002	0.021 ± 0.001	0.013 ± 0.001
^99m^Tc-temazepam NLC-1	Blood	0.036 ± 0.004	0.050 ± 0.003	0.043 ± 0.005	0.040 ± 0.006	0.035 ± 0.007
^99m^Tc-temazepam suspension	Brain	0.020 ± 0.007	0.025 ± 0.004	0.035 ± 0.003	0.019 ± 0.004	0.012 ± 0.004
^99m^Tc-temazepam NLC-1	Brain	0.040 ± 0.002	0.070 ± 0.003	0.053 ± 0.007	0.045 ± 0.005	0.040 ± 0.004
^99m^Tc-temazepam suspension	Brain/blood	0.90	0.69	1.09	0.90	0.92
^99m^Tc-temazepam NLC-1	Brain/blood	1.11	1.40	1.23	1.13	1.14

The rats were orally administered a mean of 77.5 μCi of the ^99m^Tc-temazepam suspension or the ^99m^Tc-temazepam NLC-1 formulation. The radioactivity was measured in percentage of the administered dose per gram. Data are expressed as mean ± S.D.

**Table 6:** Pharmacokinetic and targeting parameters in plasma and brain following oral administration of ^99m^Tc-temazepam suspension and ^99m^Tc-temazepam NLC-1 formulation.

Formulation	Organ	*C*_max_ (%id/g)	*T*_max_	AUC_0–t_	AUC_0–∞_	MRT_0-inf_obs_ (h)	Cl/F__obs_ (µci/%id/g/h)	*t*_1/2_ (h)
			(h)	(%id/g*h)	(%id/g*h)			
^99m^Tc-temazepam suspension	Blood	0.039 ± 0.001	1.3 ± 0.5	0.14 ± 0.02	0.23 ± 0.11	4.1 ± 0.4	467.5 ± 21.7	2.5 ± 0.4
^99m^Tc-temazepam NLC-1	Blood	0.057 * ± 0.012	1.3 ^ns^ ± 0.6	0.23 * ± 0.04	0.68 * ± 0.25	13.4 * ± 5.2	145.8 **** ± 16.4	8.9 * ± 3.5
^99m^Tc-temazepam suspension	Brain	0.033 ± 0.006	2.0 ± 0.2	0.13 ± 0.03	0.19 ± 0.08	4.9 ± 1.5	532.8 ± 52.7	2.3 ± 0.3
^99m^Tc-temazepam NLC-1	Brain	0.070 *** ± 0.001	1.0 ^***^ ± 0.1	0.27 ** ± 0.04	0.65 *** ± 0.01	10.8 * ± 2.5	119.9 **** ± 1.9	7.3 * ± 2.0
Re	3.38							
Ce	2.12							
Drug-targeting index (DTI)	1.16							

*C*_max_; peak plasma and brain concentrations, (*T*_max_); time to reach peak plasma and brain concentration, AUC_0→t_; area under the plasma and brain concentration-time curve up to definite time, AUC_0→∞_; area under the plasma and brain concentration-time curve up to time infinity, MRT; mean residence time, CL; plasma clearance and (*t*_1/2_); half-life. Data are expressed as mean ± S.D. * Significantly different from ^99m^Tc-temazepam suspension control (*p* < 0.05). ** Very significantly different from ^99m^Tc-temazepam suspension control (*p* < 0.01). *** Extremely significant different from ^99m^Tc-temazepam suspension control (*p* < 0.001). **** Extremely significant different from ^99m^Tc-temazepam suspension control (*p* < 0.0001). ^ns^ Non-significant difference compared to ^99m^Tc-temazepam suspension control (*p* > 0.05). The statistical significance was calculated by student’s *t*-test.
